# Supplementary material for: Reduction midpoint potential of a paradigm light–oxygen–voltage receptor and its modulation by methionine residues
Source: RSC Chem Biol. 2024 Apr 9;5(6):530–43. doi: 10.1039/d4cb00056k (PMC11151830; doi:10.1039/d4cb00056k)
Supplement: CB-005-D4CB00056K-s001 [file CB-005-D4CB00056K-s001.pdf]

Electronic Supplementary Material (ESI) for RSC Chemical Biology.
This journal is © The Royal Society of Chemistry 2024

Reduction Midpoint Potential of a Paradigm Light-Oxygen-Voltage Receptor and its Modulation by Methionine Residues

Andrés García de Fuentes & Andreas Möglich

Supplementary Material

Supplementary Figure 1

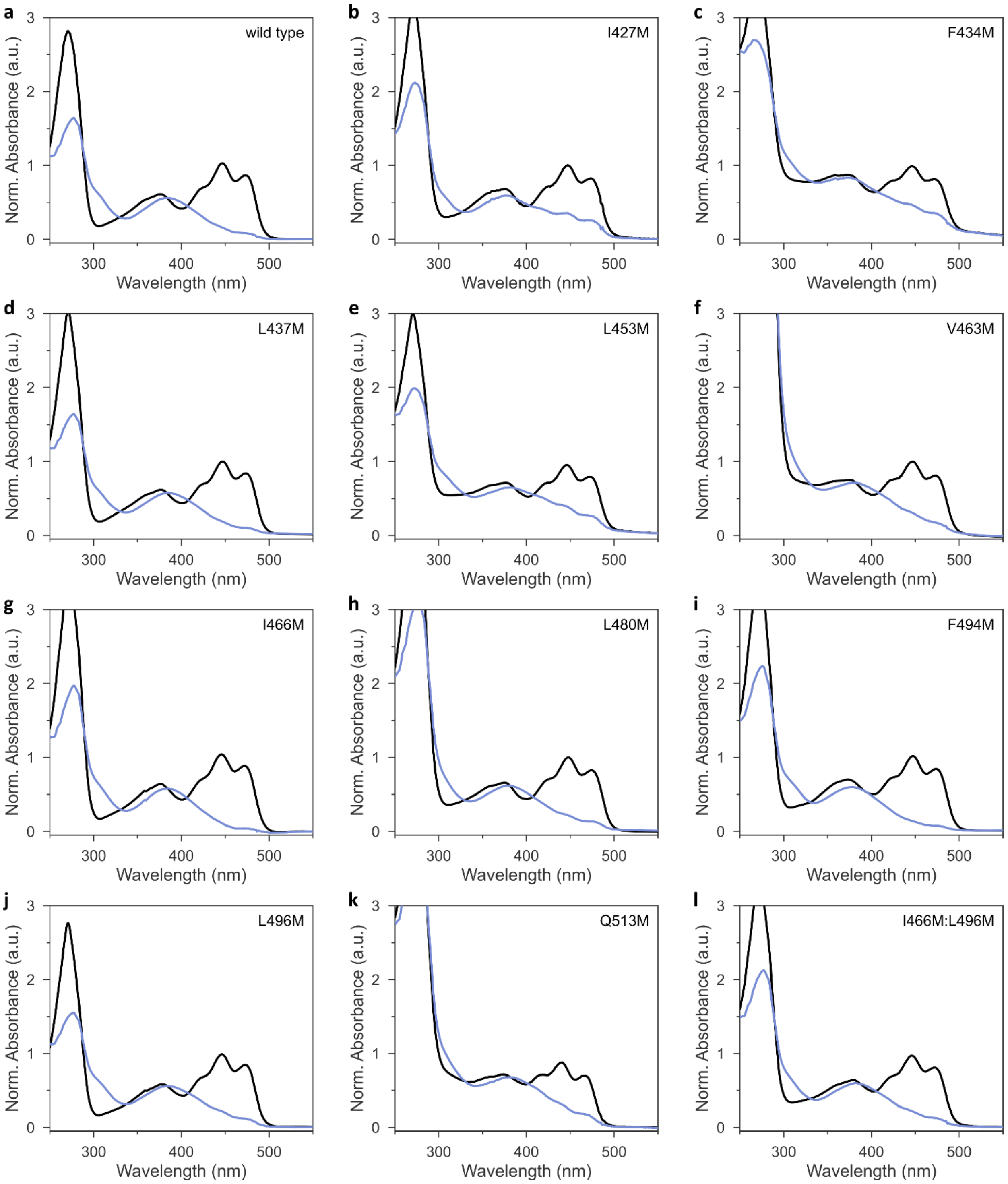

UV-vis absorbance spectra of the *As*LOV2 variants in their dark-adapted (black lines) and light-adapted states (blue). **a**, *As*LOV2 wild type. **b**, *As*LOV2 I427M. **c**, *As*LOV2 F434M. **d**, *As*LOV2 L437M. **e**, *As*LOV2 L453M. **f**, *As*LOV2 V463M. **g**, *As*LOV2 I466M. **h**, *As*LOV2 L480M. **i**, *As*LOV2 F494M. **j**, *As*LOV2 L496M. **k**, *As*LOV2 Q513M. **l**, *As*LOV2 I466M:L496M.

Supplementary Figure 2

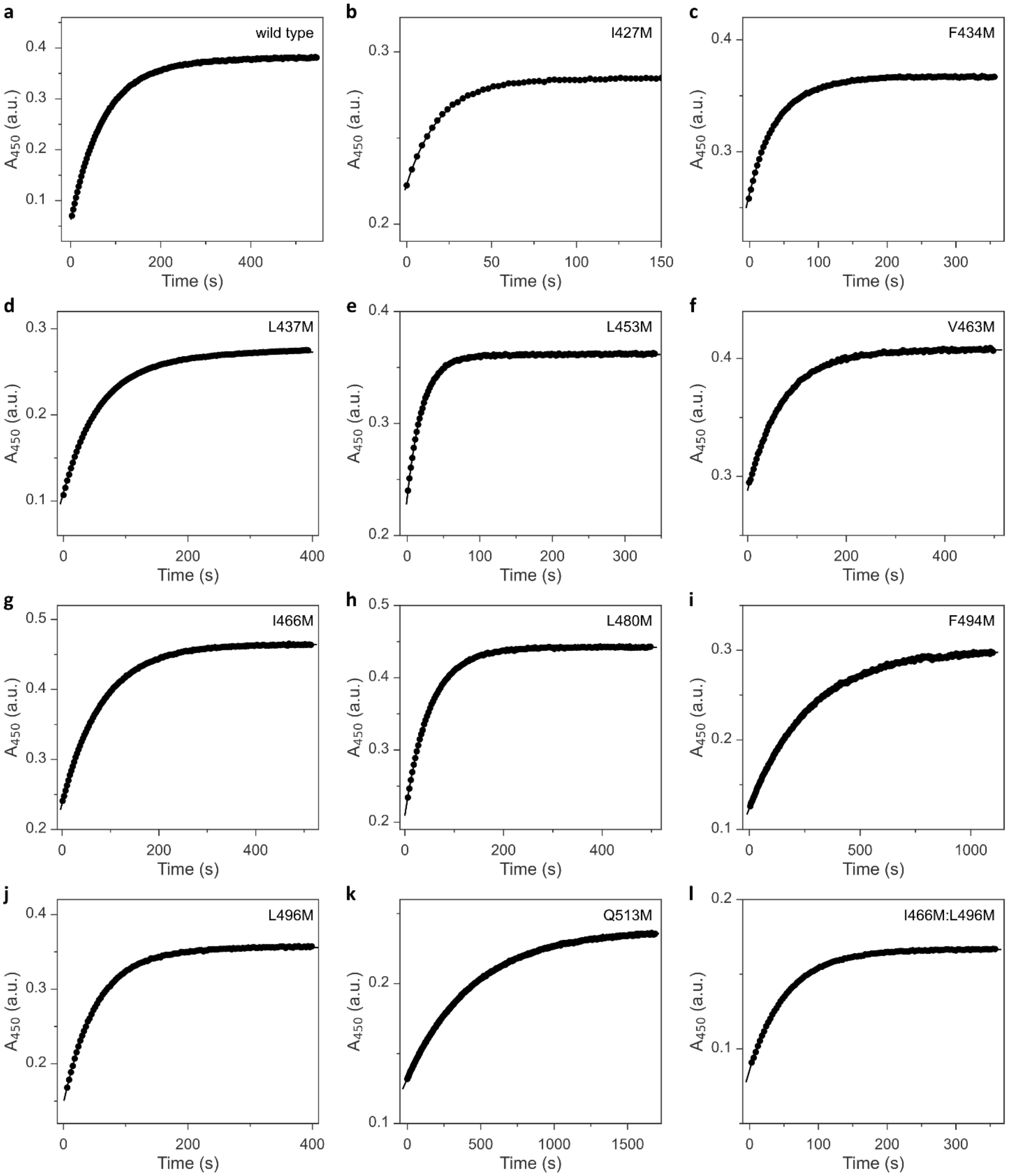

Dark-recovery kinetics of the *As*LOV2 variants monitored by absorbance measurements at 450 nm. **a**, *As*LOV2 wild type. **b**, *As*LOV2 I427M. **c**, *As*LOV2 F434M. **d**, *As*LOV2 L437M. **e**, *As*LOV2 L453M. **f**, *As*LOV2 V463M. **g**, *As*LOV2 I466M. **h**, *As*LOV2 L480M. **i**, *As*LOV2 F494M. **j**, *As*LOV2 L496M. **k**, *As*LOV2 Q513M. **l**, *As*LOV2 I466M:L496M.

 This journal is © The Royal Society of Chemistry 2024

Supplementary Figure 3

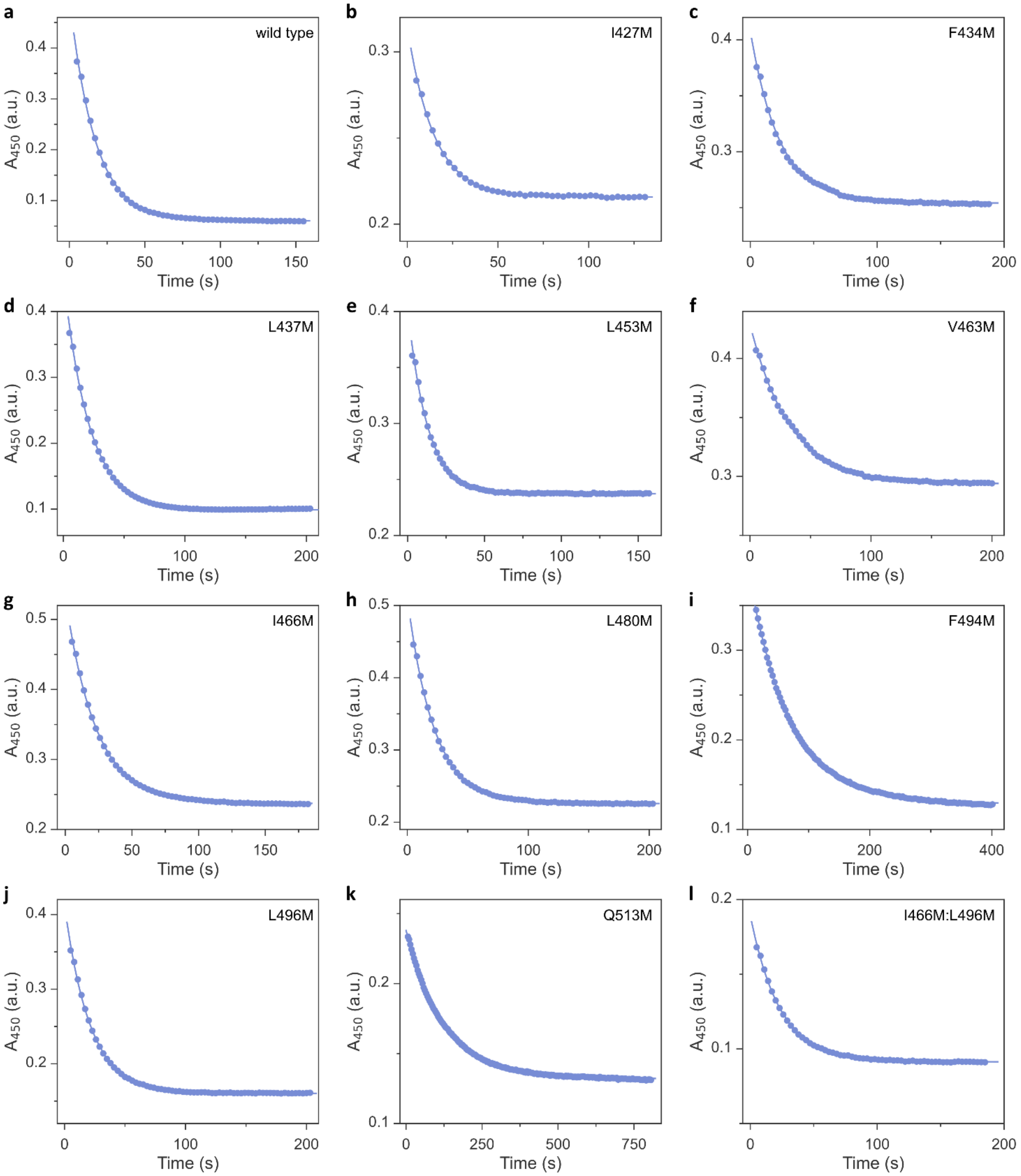

Photoactivation kinetics of the *As*LOV2 variants monitored by absorbance measurements at 450 nm. A set illumination geometry and intensity (3 mW cm^{-2}) was used in all measurements. **a**, *As*LOV2 wild type. **b**, *As*LOV2 I427M. **c**, *As*LOV2 F434M. **d**, *As*LOV2 L437M. **e**, *As*LOV2 L453M. **f**, *As*LOV2 V463M. **g**, *As*LOV2 I466M. **h**, *As*LOV2 L480M. **i**, *As*LOV2 F494M. **j**, *As*LOV2 L496M. **k**, *As*LOV2 Q513M. **l**, *As*LOV2 I466M:L496M.

This journal is © The Royal Society of Chemistry 2024

Supplementary Figure 4

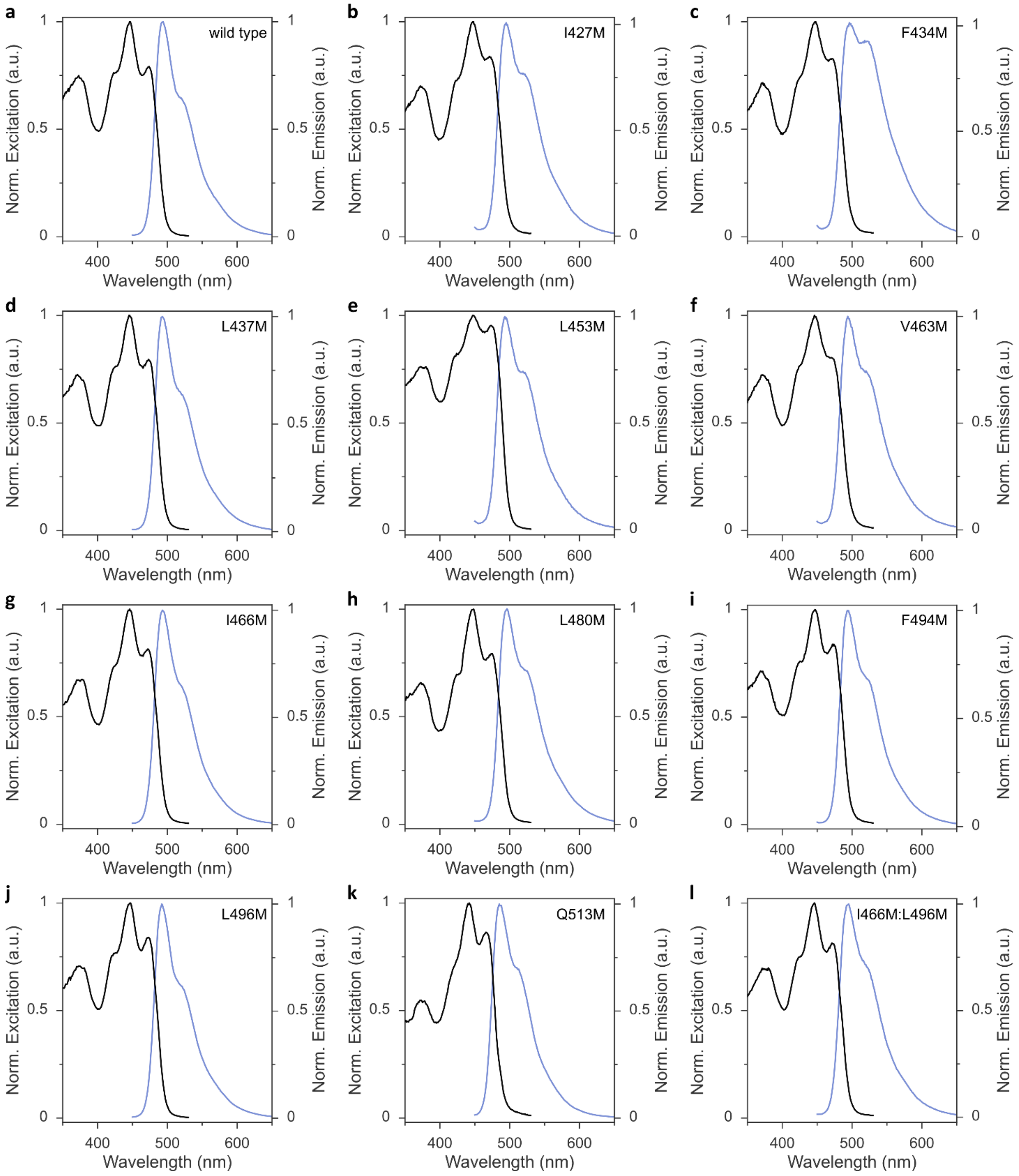

Fluorescence excitation (black lines) and emission spectra (blue) of the *As*LOV2 variants. **a**, *As*LOV2 wild type. **b**, *As*LOV2 I427M. **c**, *As*LOV2 F434M. **d**, *As*LOV2 L437M. **e**, *As*LOV2 L453M. **f**, *As*LOV2 V463M. **g**, *As*LOV2 I466M. **h**, *As*LOV2 L480M. **i**, *As*LOV2 F494M. **j**, *As*LOV2 L496M. **k**, *As*LOV2 Q513M. **l**, *As*LOV2 I466M:L496M.

 This journal is © The Royal Society of Chemistry 2024

Supplementary Figure 5

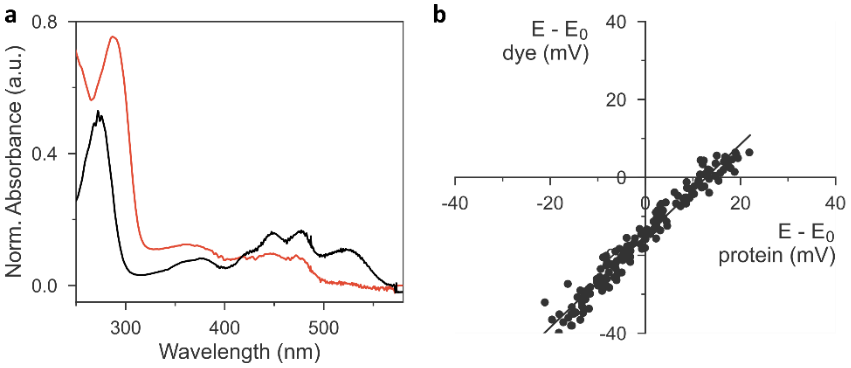

Determination of the flavin reduction midpoint potentials in *As*LOV2 wild type by the xanthine-oxidase (XO) method. **a**, Absorbance spectra at the start of the XO reduction kinetics (black lines) and after 5 hours (red). **b**, The correlation of the oxidized and reduced fractions of the redox indicator phenosafranine and *As*LOV2 according to the Nernst equation allows the determination of the flavin reduction midpoint potential in *As*LOV2. The *y* axis denotes $E - E_{0,r}$ for the dye, and the *x* axis plots the corresponding values $E - E_0$ for the LOV protein.

Supplementary Figure 6

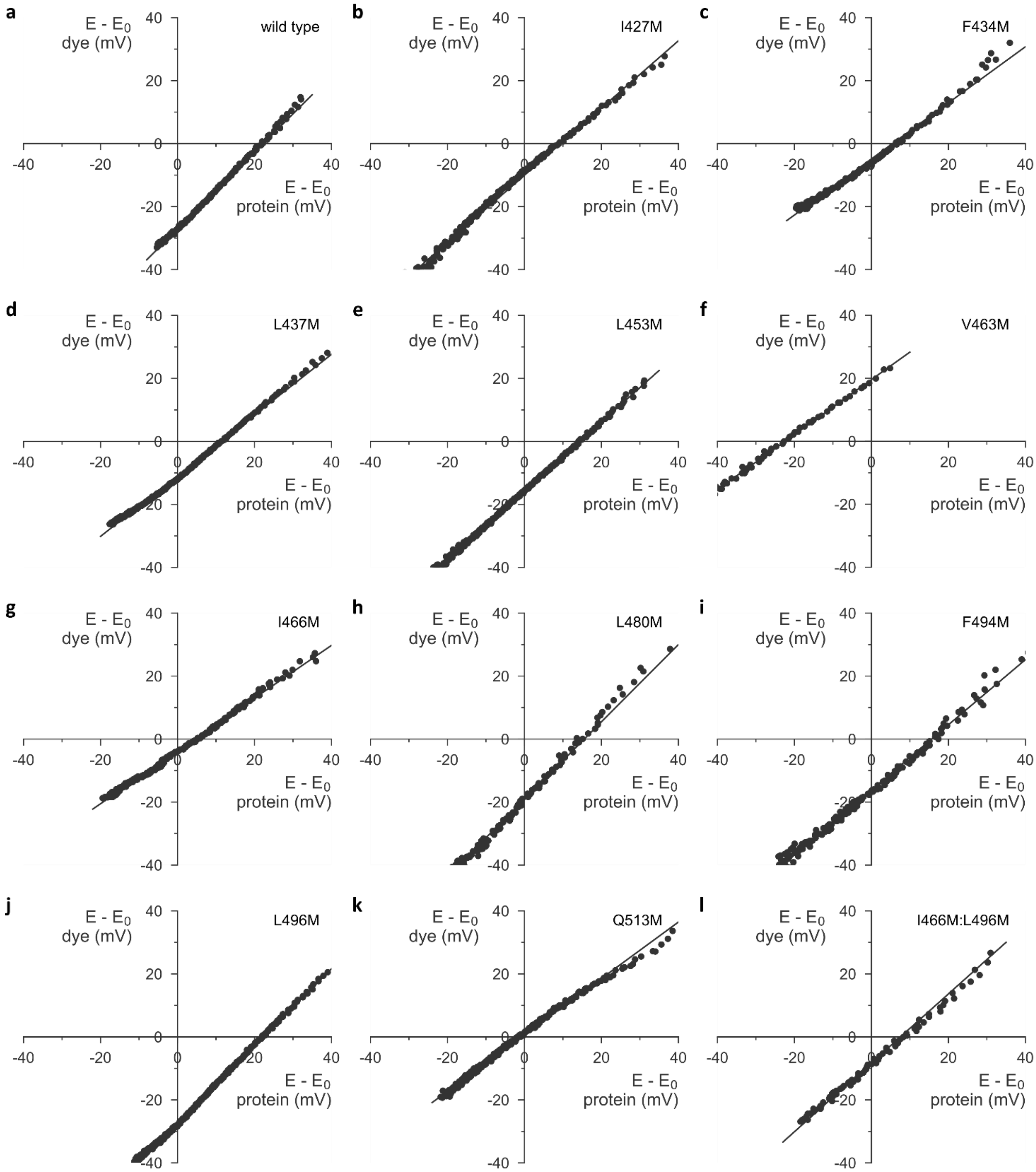

Determination of the flavin reduction midpoint potentials in the *As*LOV2 variants. The *y* axis denotes $E - E_{0,\mathrm{r}}$ for the dye, and the *x* axis plots the corresponding values $E - E_0$ for the LOV protein. **a**, *As*LOV2 wild type. **b**, *As*LOV2 I427M. **c**, *As*LOV2 F434M. **d**, *As*LOV2 L437M. **e**, *As*LOV2 L453M. **f**, *As*LOV2 V463M. **g**, *As*LOV2 I466M. **h**, *As*LOV2 L480M. **i**, *As*LOV2 F494M. **j**, *As*LOV2 L496M. **k**, *As*LOV2 Q513M. **l**, *As*LOV2 I466M:L496M.

This journal is © The Royal Society of Chemistry 2024

Supplementary Figure 7

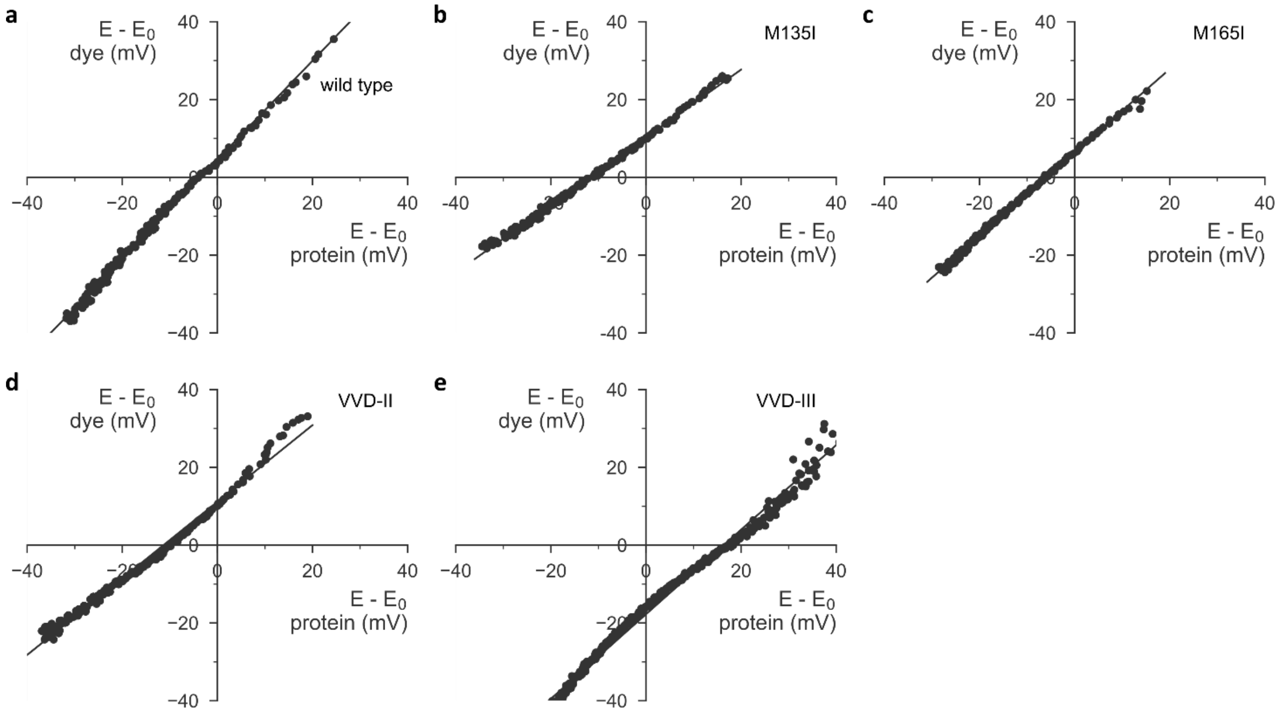

Determination of the flavin reduction midpoint potentials in the indicated *Nc*VVD variants. The *y* axis denotes $E - E_{0,r}$ for the dye, and the *x* axis plots the corresponding values $E - E_0$ for the LOV protein. **a**, *Nc*VVD wild type. **b**, *Nc*VVD M135I. **c**, *Nc*VVD M165I. **d**, *Nc*VVD-II (M135I:M165I). **e**, *Nc*VVD-III (C108A:M135I:M165I).

Supplementary Figure 8

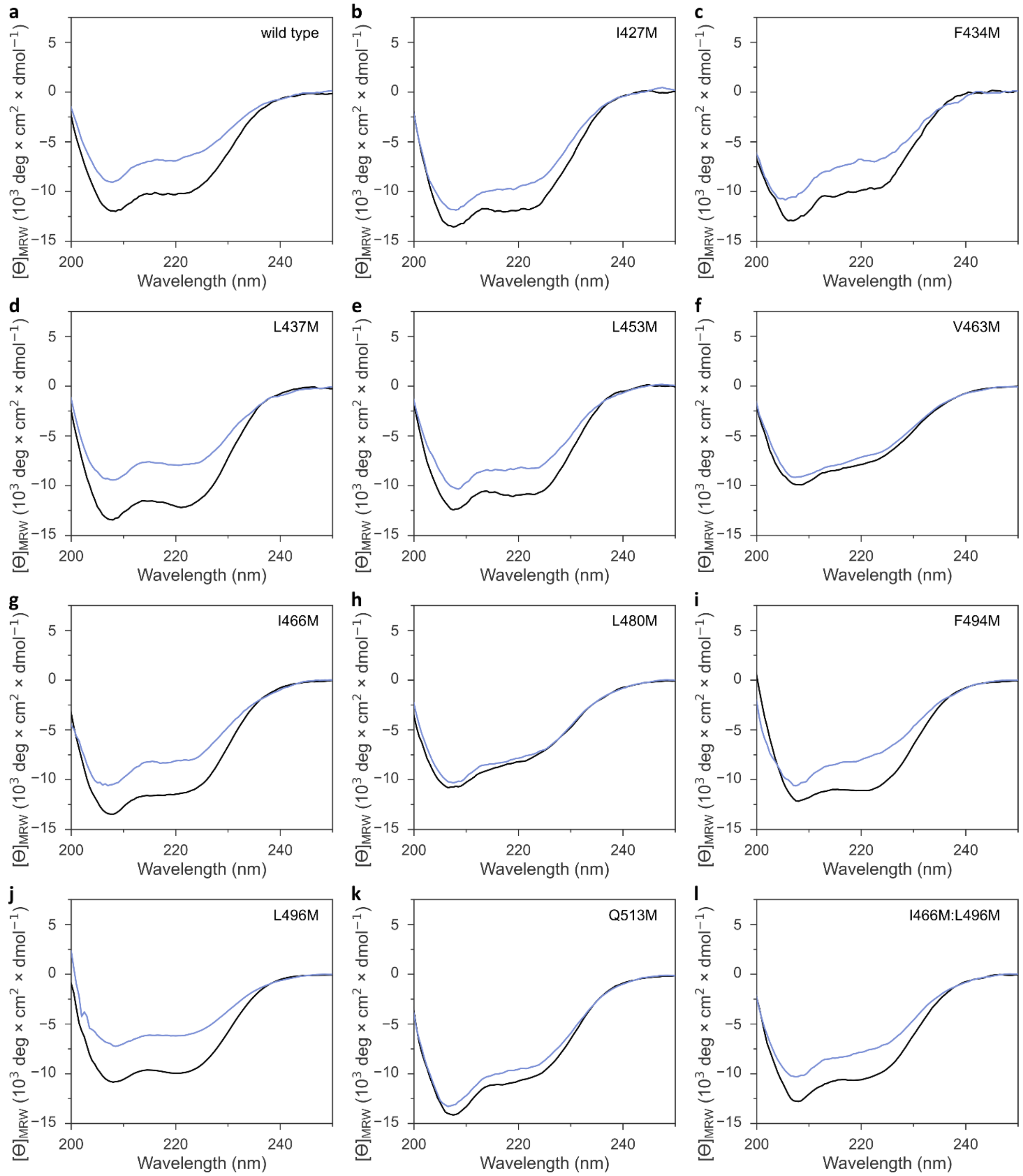

Far-UV circular dichroism spectra of the *As*LOV2 variants in their dark-adapted (black lines) and light-adapted states (blue). **a**, *As*LOV2 wild type. **b**, *As*LOV2 I427M. **c**, *As*LOV2 F434M. **d**, *As*LOV2 L437M. **e**, *As*LOV2 L453M. **f**, *As*LOV2 V463M. **g**, *As*LOV2 I466M. **h**, *As*LOV2 L480M. **i**, *As*LOV2 F494M. **j**, *As*LOV2 L496M. **k**, *As*LOV2 Q513M. **l**, *As*LOV2 I466M:L496M.

This journal is © The Royal Society of Chemistry 2024
